# Family member and service provider experiences and perspectives of a digital surveillance and service navigation approach in multicultural context: a qualitative study in identifying the barriers and enablers to Watch Me Grow-Electronic (WMG-E) program with a culturally diverse community

**DOI:** 10.1186/s12913-024-11397-y

**Published:** 2024-08-24

**Authors:** Karlen R. Barr, Patrick Hawker, Teresa Winata, Si Wang, Melissa Smead, Hilda Ignatius, Jane Kohlhoff, Virginia Schmied, Bin Jalaludin, Kenny Lawson, Siaw-Teng Liaw, Raghu Lingam, Andrew Page, Christa Lam-Cassettari, Katherine Boydell, Ping-I Lin, Ilan Katz, Ann Dadich, Shanti Raman, Rebekah Grace, Aunty Kerrie Doyle, Tom McClean, Blaise Di Mento, John Preddy, Susan Woolfenden, Valsamma Eapen, the Watch Me Grow-Electronic (WMG-E) study group, S. T. Liaw, Daniel P. Lin, Rebecca Grace, Sara Cibralic, Anthony Mendoza Diaz, Jodie Bruce, Nicole Myers, Joseph Descallar, Cathy Kaplun, Amit Arora, Victoria Blight, Angela Wood

**Affiliations:** 1 Academic Unit of Infant, Child and Adolescent Psychiatry, South Western Sydney Local Health District, Liverpool, NSW Australia; 2 Discipline of Psychiatry and Mental Health, Faculty of Medicine and Health, University of New South Wales, Sydney, NSW Australia; 3 Ingham Institute for Applied Medical Research, Liverpool, NSW Australia; 4 National Disability Insurance Scheme Quality and Safeguards Commission, Parramatta, NSW Australia; 5 Research and Evaluation Group, The Salvation Army, Sydney, NSW Australia; 6 Murrumbidgee Local Health District, Wagga Wagga, NSW Australia; 7 South Western Sydney Local Health District, Liverpool, NSW Australia; 8 Karitane, Carramar, NSW Australia; 9 School of Nursing and Midwifery, Western Sydney University, Parramatta, NSW Australia; 10 School of Public Health and Community Medicine, Faculty of Medicine and Health, University of New South Wales, Sydney, NSW Australia; 11 School of Business, Western Sydney University, Campbelltown, NSW Australia; 12 WHO Collaborating Centre for eHealth, University of New South Wales, Sydney, NSW Australia; 13 Population Child Health Research Group, School of Women’s and Children’s Health, Faculty of Medicine, University of New South Wales, Sydney, NSW Australia; 14 School of Medicine, Western Sydney University, Parramatta, NSW Australia; 15 Black Dog Institute, Sydney, NSW Australia; 16 Social Policy Research Centre, Faculty of Arts, Design, and Architecture, University of New South Wales, Sydney, NSW Australia; 17 Transforming Early Education and Child Health Research Centre, Western Sydney University, Campbelltown, NSW Australia; 18 Uniting, Parramatta, NSW Australia; 19 Rural Clinical School, School of Clinical Medicine, University of New South Wales, Wagga Wagga, NSW Australia; 20 Sydney Medical School, Faculty of Medicine and Health, University of Sydney, Sydney, NSW Australia; 21 Sydney Local Health District, Sydney, NSW Australia

**Keywords:** Child development, Multicultural families, Developmental checks, Digital developmental surveillance, Service navigator

## Abstract

**Background:**

Children and families from priority populations experienced significant psychosocial and mental health issues to the COVID-19 pandemic. Yet they also faced significant barriers to service access, particularly families from culturally and linguistically diverse (CALD) backgrounds. With most child and family health nurse clinics ceasing in-person consultations due to the pandemic, many children missed out on health and developmental checks. The aim of this study was to investigate the perspectives and experiences of family members and service providers from an urban, CALD community regarding the implementation of a digital, developmental surveillance, Watch Me Grow-Electronic (WMG-E) program.

**Methods:**

Semi-structured interviews were conducted with 17 family members, service navigators, and service providers in a multicultural community in South Western Sydney, Australia. This qualitative study is an implementation evaluation which formed as part of a larger, two-site, randomised controlled trial of the WMG-E program. A reflexive thematic analysis approach, using inductive coding, was adopted to analyse the data.

**Results:**

Participants highlighted the comprehensive and personalised support offered by existing child and family health services. The WMG-E was deemed beneficial because the weblink was easy and quick to use and it enabled access to a service navigator who support family access to relevant services. However, the WMG-E was problematic because of technology or language barriers, and it did not facilitate immediate clinician involvement when families completed the weblink.

**Conclusions:**

Families and service providers in this qualitative study found that using WMG-E empowered parents and caregivers to access developmental screening and learn more about their child’s development and engage with relevant services. This beds down a new and innovative solution to the current service delivery gap and create mechanisms that can engage families currently not accessing services, and increases knowledge around navigating the health and social care services. Notwithstanding the issues that were raised by families and service providers, which include accessibility challenges for CALD communities, absence of clinical oversight during screening, and narrow scope of engagement with available services being offered, it is worth noting that improvements regarding these implementation factors must be considered and addressed in order to have longevity and sustainability of the program.

Trial registration.

The study is part of a large randomised controlled trial (Protocol No. 1.0, Version 3.1) was registered with ANZCTR (registration number: ACTRN12621000766819) on July 21st, 2021 and reporting of the trial results will be according to recommendations in the CONSORT Statement.

**Supplementary Information:**

The online version contains supplementary material available at 10.1186/s12913-024-11397-y.

## Background

People from culturally and linguistically diverse (CALD) backgrounds in Australia are known to experience multiple social disadvantages and face challenges in health and health care needs [1]. Previous reviews on access to health services among CALD populations have focused on specific health issues [2–4]. However, there is a dearth of evidence available on child and family health service-related problems in CALD populations. Recent research and government policies have increasingly focused on early intervention for child developmental delays in the general population [5–7]. It is understood that children who do not access early developmental screening are at-risk for delays in early identification of developmental issues and delayed access to appropriate interventions, leading to future difficulties [8]. For instance, one in five Australian children starting school do not have the developmental skills necessary to thrive [9]. In Australia, each state and territory has its own processes around developmental surveillance; in most cases, this involves developmental screening completed by Child and Family Health Nurses at 6, 12, 24, 36, and 48 months. While there are state and federal developmental surveillance models (see Table 1), the uptake is variable, especially for children from CALD backgrounds. These children are at higher developmental risk, but the developmental concerns/delays go unidentified until the start of school in many instances. This impact was further increased by the COVID-19 pandemic effect (e.g. service closures and resource constraints), thereby missing out on early screening and intervention opportunities [10].

**Table 1:** Current Australian national and NSW state-based models of child development surveillance, screening and diagnosis [11]

Model feature	NSW	Australia
Target population – universal	√	√
Progressive or proportionate universalism^a^	√	√
Ages for contact	Birth to 4 years	Birth to 5 years
Number of contact points	8	Not specified
Settings	Home visits & clinics	Home visits & clinics
Primary^b^ Healthcare professionals involved Secondary^c^	Child and family health nurses, midwives, GPs – Paediatricians, social workers, psychologists, speech pathologist, and so forth	Child and family health nurses, midwives, GPs – Paediatricians, social workers, psychologists, speech pathologist, and so forth
Physical health monitoring^d^	√	√
Hearing and vision screening^e^	√	√
Growth monitoring^d^	√	√
Health promotion	√	√
Developmental assessment^f^	√	√
Child developmental screening tool	Blue Book (Learn the Signs. Act Early [LTSAE])	Based on jurisdiction
Immunisation	√	√
Anticipatory guidance		√
Autism screening	X	X
IT utilised in program	X	X

^a^ Each of the comprehensive models sought to include all children (universal reach) although most noted a need for targeted resources for disadvantaged children. Documents varied with respect to the amount of detail regarding the identification, engagement and management of such disadvantaged children, although sub-populations of indigenous/aboriginal families, teenage mothers and children living in poverty were frequently mentioned as being prioritised

^b^ Primary health care is provided by General Practitioners who serve at the entry level to the health system and, as such, is usually a person’s first encounter with the health system. They provide a broad range of activities and services, from health promotion and prevention, to treatment and management of acute and chronic conditions

^c^ Secondary health care relates to a specialist medical practitioner (e.g. paediatricians) when patients are referred from a primary care service such as the General Practice to the next level in the service system, and this could be in a hospital or a community based specialist clinic

^d^ Monitoring enables ongoing tracking of health and developmental problems as well as linking children with services for further assessment or intervention when concerns are identified

^e^ Developmental screening is a test (or a series of tests) performed on a population to systematically examine whether the child is meeting the developmental milestones, with a view of identifying any developmental delay/problems early

^f^ Developmental assessments are a comprehensive evaluation of a child’s physical, intellectual, language, social and emotional development. It is the process of mapping a child’s performance compared with children of similar age, and aims to highlight what normal developmental parameters are, when and how to assess a child, and when to refer for specialist assessment

Children from CALD or socioeconomically disadvantaged communities have an increased risk of developmental challenges [12, 13]. According to the ‘inverse care law,’ children from socio-economically disadvantaged and CALD backgrounds and who are most at-risk for developmental delays are less likely and less able to access preventative care services, such as developmental surveillance programs [14, 15]. While such programs aim to promote child health and development, and to facilitate early detection and intervention, including growth monitoring, physical health, developmental surveillance, and health promotion, data have suggested that uptake of these programs has been low [11]. In a study involving a 2000-strong cohort in South Western Sydney [16], for example, it was found that while up to 30% of children are at developmental risk by their 18-month ‘well-child’ check, only 30–50% of these children attending primary healthcare have their developmental surveillance record completed [17–19]. Another study [20] highlighted the need for practice and policy changes including training of general practitioners in developmental surveillance, as well as allocation of time and financial remuneration for developmental checks in primary care [1]. Qualitative findings from previous research have demonstrated enabling factors such as proximity, continuity of care, opportunistic contacts, integrated/connected engagement between services, as well as parental and service provider language and cultural concordance [21–23].

Accessing services might be particularly difficult for CALD families due to their cultural beliefs, language barriers, poor health literacy and awareness of services, and the complex nature of child and family health services [14, 21, 23, 24]. The COVID-19 pandemic might have exacerbated difficulties with service access for CALD families [25]. These accessibility challenges for families from CALD backgrounds extend to telehealth or online services due to few adaptations and translations [26]. While the use of web-based applications can increase health screening uptake, more needs to be understood about real-world implementation [27].

Increasingly, family member perspectives are being included during the evaluation of health initiatives [21, 23, 28]. Qualitative research that focuses on in-depth description and personal experience stories and gathers the perspectives of family members and service providers on developmental surveillance and service access is important to provide insights around what works and how developmental surveillance monitoring can be improved.

## Research aims

As part of the entire implementation research trial, the aim of this study is therefore to qualitatively evaluate the engagement with developmental surveillance, services access to child and family health, and uptake of service recommendations by identifying the barriers and enablers of our digital developmental surveillance program, WMG-E, according to our CALD family members, service navigators and service providers. Specifically, we focused on perspectives regarding the developmental surveillance program and the service navigator role in a multicultural community.

To address our research aims of this qualitative study, we formulated the following research questions:

1. What are the perceived barriers and enablers to family engagement in relation to the current Child and Family Health Services model in a CALD/multicultural community, as reported by family members, service navigators, and service providers?
2. How does the implementation of the WMG-E digital surveillance (weblink) impact access to child and family health services, and what are the key factors influencing the uptake of service recommendations among multicultural families, according to the perspectives of family members, service navigators, and service providers?
3. What roles do service navigators play in facilitating engagement with the WMG-E program and improving service access and uptake of recommendations, and what challenges and strategies do they encounter in a multicultural community context?

## Methods

### Study context

This qualitative study was part of a randomised control trial (RCT) to evaluate our health services implementation research trial – the Watch Me Grow-Electronic (WMG-E) digital surveillance approach and a service navigation component during the COVID-19 pandemic [29]. This RCT occurred in two sites – Murrumbidgee, New South Wales, and South Western Sydney (SWS), New South Wales, Australia. The WMG-E program includes a digital weblink whereby families are screened for developmental issues, parental mental health problems, and psychosocial concerns. Following completion of this screener, families were randomised into a treatment group where they were supported by a service navigator or a usual care control group.

Our research team has decided to report our findings separately by site, as there are distinct implications and needs being put forward and addressed by the questions and responses relevant to each site contexts – for instance, certain language and cultural factors may be more common to the urban, multicultural site; hence, issues and enablers around these factors must be addressed and discussed further into their health and social care system specific to CALD populations; whereas workforce and demand in resources covering the large geographical areas of the rural/remote site may be put to light and therefore need more investigations and discussions.

### The program: Watch Me Grow-Electronic (WMG-E)

The Watch Me Grow-Electronic (WMG-E) is an innovative digital surveillance solution which comprised of a pragmatic, implementation trial that was piloted in two distinct populations (urban multicultural and rural/regional sites) using opportunistic service contacts within the community with automated reminders for ongoing monitoring [29]. Specifically, the WMG-E platform is a digital application in the form of a weblink, developed to help services reach vulnerable families, including those in CALD communities [26, 29]. WMG-E assesses child development, as well as family psychosocial and parental mental health needs, and is available in four languages, including English, Mandarin, Vietnamese and Arabic. WMG-E includes the Learn the Signs Act Early (LTSAE) tool, developed by the Centers for Disease Control and Prevention (CDC), to provide age-based developmental checks for children from birth to age five years [30]. Parents are encouraged to actively participate in their child’s developmental screening through WMG-E, using opportunistic service contacts, such as routine health contacts or via a link that a trusted service provider issues to families at home and in the community. Once engaged, at the next recommended ages when developmental checks are due, automated reminder emails or mobile text messages are sent to parents to ensure ongoing monitoring. If developmental concerns are raised, parents are informed and assisted to seek further assessment by health professionals.

In addition to WMG-E, service navigators are incorporated as part of the roll-out intervention to assist families to navigate and access services [30, 31]. Service navigators are trained to use their knowledge of health, mental health, and social care services, as well as the professionals in the community to help families before, during, and after service use. Service navigators consider a family’s individual needs and facilitate a connection between families and relevant services [32]. When needs were identified, service navigators supported families on a case-by-case basis to link them with the relevant services and resources, as required. The service navigator builds working relationships, solves problems, and supports families while the families learn to self-navigate the health system. The introduction of service navigators within health systems is relatively new in Australia [33–35], and research is required to understand family experiences with service navigators.

### Participants in this qualitative study

The participants involved in this qualitative study were recruited from our major WMG-E trial cohort that make up the CALD urban community/population in South Western Sydney, New South Wales, with approximately 80% of households primarily communicating in a language other than English [36]. Convenience sampling was used to invite family members to participate, whereby a quarter of multicultural family members (*n* = 14 out of a total of 66 multicultural families in SWS) who participated in the larger RCT were invited to participate in an interview. Similarly, service provider participants were recruited using convenience sampling from the WMG-E trial; whereby they were then invited to participate in this interview study, alongside service navigators who were involved in the project. Information about this qualitative research and an invitation to participate was sent to potential participants via email or text message. Overall, our participants included ten family members, five service providers and two service navigators (*n* = 17) that participated in this interview study.

### Characteristics of participants involved in this qualitative study

Ninety percent of the family member participants were females; with 40% aged 36 to 40 and the remaining equally distributed in the age groups 31 to 35 and 26 to 30. All participants had attained education to the level of high school (40%) or above, with 30% having a bachelor and 20% a post graduate degree and 10% a Graduate Diploma. Forty percent were of Asian background, 20% Australian and the rest were from Middle Eastern and Pacific backgrounds. English was the most commonly spoken language at home (60%) followed by Vietnamese (20%). Service provider participants were all females with 60% from NGO and the remaining from Government organisations (40%). Sixty percent were in Management and the rest in Allied Health roles. Service navigators were all females.

### Interview recruitment and procedures

This study was approved by the South Western Sydney Local Health District Human Research Ethics Committee (HREC reference 2020/ETH01418), and all participants provided written informed consent. All interviews were conducted online with participants.

Semi-structured interviews were conducted by two researchers, one person within and one external to the research (TW and HI), guided by an interview guide (see Supplementary File-Appendix 1). Family members were presented with five questions about their experiences of attending child and family health services, the impacts of COVID-19 on their appointments with child and family health services, and experiences with the WMG-E platform. Family members that were part of the intervention group answered an additional two questions based on their experience with the service navigator. Service providers and service navigators were asked ten questions on their experiences of providing developmental checks, their involvement in the WMG-E program, the benefits of or limitations with the WMG-E weblink, and their perceptions of the WMG-E weblink for developmental checks and the service navigator. Interview duration ranged between twenty and sixty minutes. The interviews were audio-recorded, transcribed verbatim, and identifying information was removed. Consumer participants were provided with a gift voucher in lieu of their time for participation.

### Data analysis

**Fig. 1:**
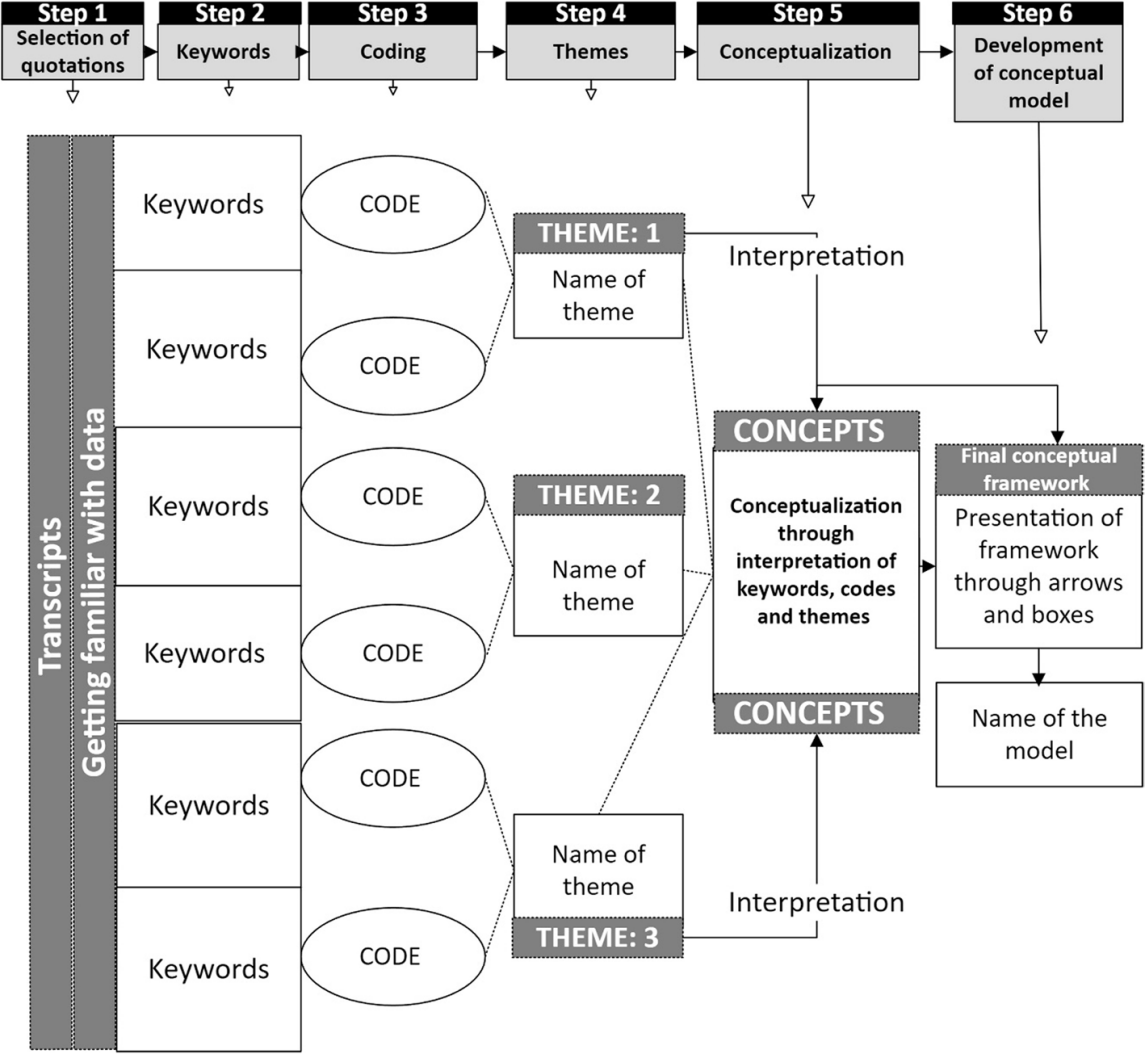
A systematic thematic analysis process: A novel six-step process for conceptual model development in qualitative research [42]

All interviews were transcribed by professional transcription services and coded using NVivo12 [37]. In utilising an reflexive thematic analysis underpinned by grounded theory [38], the research team has followed a rigorous, systematic six-step qualitative analytical process based on Braun and Clarke [39]’s thematic analysis (as according to Fig. 1) that leads to the development of a conceptual model in order to triangulate all collected data by cross-checking and agreeing/disagreeing on the themes and subthemes that have emerged from the qualitative interviews [39]. To do this, transcribed interviews were initially coded by two qualitative researchers (KRB, PH). As these researchers were also members of the study team, a randomly selected set of interview transcripts were also selected and coded by an external reviewer (HI) and themes were compared. Any disagreements were resolved through discussions until consensus was reached on themes and subthemes. This system of coding provided an opportunity for identifying a consensus based themes and subthemes of individual experiences and perceptions regarding the feasibility of participating and utilising our digital screening, monitoring and navigation program through WMG-E [40]. Thematic analysis was undertaken to develop key themes/subthemes relating to parental/caregivers and service providers/navigators’ experiences and perceptions; which was done by inductive coding, [38, 39] allowing data to be organized and used to explore connections between data elements and to develop conceptual items. Once coded, segments of data were then linked in a formal fashion to allow themes to emerge and to determine relationships between different data sets. Data saturation had been reached with the current interview sample size through a strict procedure of pilot interview analysis over multiple iterations/discussions between research members. The study has been reported in line with the Standards for Reporting Qualitative Research (see Supplementary File Table 2) [41].

## Results

**Fig. 2:**
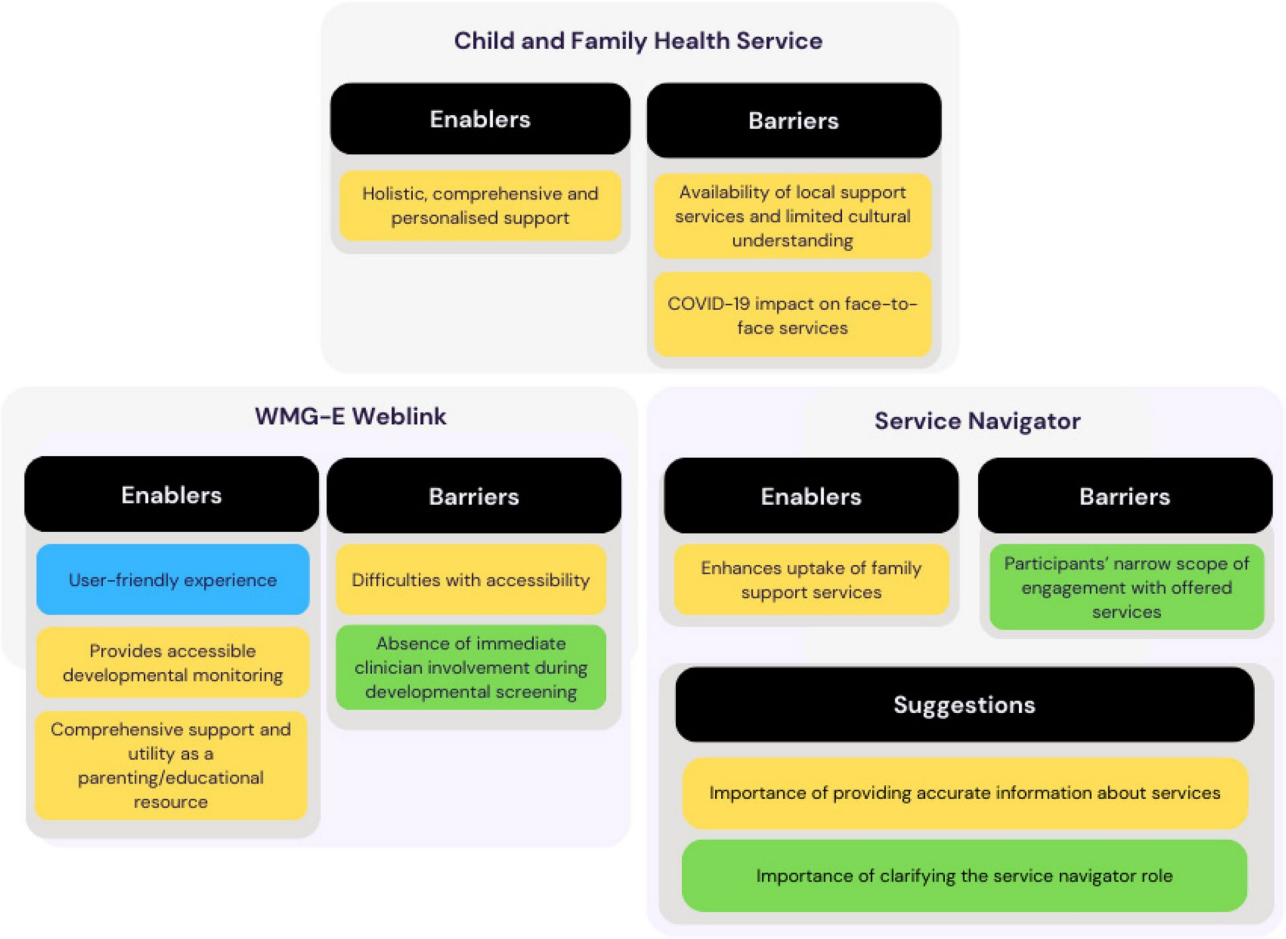
Summary of barriers, enablers, and suggested improvements from parent/caregiver as well as service providers/professionals’ perspectives around the current Child and Family Health Service (at the time), participation in the WMG-E weblink, and the Service Navigator program. Notes: Yellow = perspectives from family members, service providers and service navigators; Blue = perspectives from family members only; Green = perspectives from service providers and service navigators. WMG-E, Watch Me Grow-Electronic

The qualitative findings were separated into four themes: enablers and barriers of the current Child and Family Health Services model, enablers and barriers of the WMG-E weblink, enablers and barriers of Service Navigator implementation and suggested improvements for the Service Navigator role. To address our research questions in this qualitative study, we have explicitly connected our identified themes and subthemes (alongside supporting quotes) under each of the relevant research questions to enhance clarity and clearer structural flow/presentation of our participant data. A summary of themes and whether the themes were supported by service provider, service navigator or family member perspectives is illustrated in Fig. 2.

**Research Question 1: **What are the perceived barriers and enablers to family engagement in relation to the current child and family health services model in a CALD/multicultural community, as reported by family members, service navigators, and service providers?

## Theme 1.0: Enablers and barriers of the current child and family health services model

### Enablers

#### Holistic, comprehensive and personalised support

Participants described how identifying the families’ needs related to child and family health helps the relevant service provider or navigator to enable the families to navigate the intricate service system:

*“What we know is that the service system is quite complex and families don’t often know how to get the right service for their families, so our practitioners are very skilled in knowing what the right service would be for families”* (service provider SP02).

Service providers discussed how an in-depth evaluation of each family’s needs is conducted, allowing for individualised support:

*“When we get a referral for a family, we talk to them and complete an assessment about the individual needs, what supports they want and what goals they have for their families”* (service provider SP02).

Participants described how services provide interim case work to support families during the linkage process to long-term community services:

*“The program is able to meet the needs of most referrals because we either meet that immediate need or we are able to support them whilst they are waiting for that ongoing management”* (service provider SP05).

Beyond family engagement, the social care service provider supports families to access services for education, health, skill development programs, and other community services in the area:

*“We provide support to services around skill development, but we can also provide community development”* (service provider SP02).

Service providers discussed a swift adaptation to remote working conditions, expansion of usual criteria, and adoption of new support services in order to mitigate unprecedented challenges posed by COVID-19.

Participants described how child and family health services provide assessment of child’s social and emotional health and parental psychosocial health while also facilitating connection with community support services:

*“If we are finished care at our end, we would discharge and refer them to other services particularly within the tertiary services”* (service provider SP03).

### Barriers

#### Restricted by availability of local support services and limited cultural understanding

Some participants discussed the difficulty of accessing services in their area and how it was important for migrant families to be connected with information and services early in their child’s development:

*“I think they should be given a more clearer picture of how do we have to be because this was my first child in Australia, so it was hard for me to figure out where am I supposed to go?”* (family member FA34).

One participant shared how a clinician did not understand her culture and emphasised the importance for service providers to have cultural sensitivity so that families could build a relationship with them:

*“I like the [clinician] in [location] because they are [multicultural] like me, so very friendly talk... I think because of the people, because you know how this kind of relationship is important, especially new mum and also first baby”* (family member FA43).

#### COVID-19 restrictions and lockdowns impact on services

The largest obstacle encountered during the study was closures of clinics due to lockdowns and restrictions emplaced by the COVID-19 pandemic effect. To echo this, participants discussed prioritisation of telehealth services with substantial limitations to face-to-face services:

*“The majority of our service was switched to telehealth. We were very limited in the amount of face-to-face visits that we were allowed to attend… there had to be significant clinical risk for that home visit to be attended”* (service provider SP04).

A few service providers shared how the COVID-19 pandemic saw an increased demand for child and family health services:

*“We ended up getting 60 or 70 referrals a week, which is double what we would normally get”* (service provider SP02).

Increases in child behavioural issues and maternal mental health concerns were also described as a result of COVID-19:

*“There was definitely an increase in behavioural issues with preschoolers particularly”* (service provider SP03).

**Research Question 2: **How does the implementation of the WMG-E digital surveillance (weblink) impact access to child and family health services, and what are the key factors influencing the uptake of service recommendations among multicultural families, according to the perspectives of family members, service navigators, and service providers?

## Theme 2.0: Enablers and barriers of the WMG-E weblink

### Enablers

#### User-friendly experience

Most family members reported that the weblink was straightforward and easy to use:

*“To use [the weblink] it was really easy and convenient”* (family member FA08).

Many family members reported that the survey took a short amount of time to complete:

*“A very short amount of time. I can’t tell you exactly, but it was less than 10 minutes”* (family member FA16).

Some family members commented that the weblink provided an easy way to track their child’s development:

*“It’s really great because it will be easier for us to maybe track information and keep … everything about how to progress with the child’s health”* (family member FA34).

### Provides accessible developmental monitoring

Some participants shared how having a digital tool was helpful as it allowed parents to take initiative and could be completed by parents who had limited time:

*“Everyone has smartphones and it's the best way to engage with people... People are working more too and back to work earlier. They've got so many commitments, so yeah, just having that access via the phone is so much better”* (service navigator 1).

The weblink was described as increasing accessibility of services by providing multiple referral pathways and the option for self-referral:

*“Having many referral pathways and options is always great”* (service provider SP05).

The digital platform offered developmental screening in an accessible manner, filling gaps in traditional healthcare assessment services:

*“There is a huge gap in families being able to access developmental checks through the developmental checks at community level at primary level so I see this is definitely a facilitator I would be able to help that process”* (service provider SP03).

The weblink also offered anonymity, which might be comforting to some parents:

*“One of the benefits of using the weblink is having something that’s in the digital age that parents can quickly go onto. It’s a little bit anonymous”* (service provider SP03).

The weblink was described as aligning with technological advances in society, which could optimise health services:

*“Families want to utilise technology… sometimes it’s the rigidity of our systems that prevent these things likes apps and technology from us actually utilising them to their best”* (service provider SP04).

### Comprehensive support and utility as a parenting/educational resource

Many participants commented that the weblink provided families with information and education about their child’s development and anticipatory guidance about normative developmental milestones:

*“I guess another benefit is that it does add an extra level of education to parents that even if they are not potentially noticing red flags or the need to access further care they are getting education on what their child should be doing so I do know that it does provide normative developmental milestones which is fantastic, and it’s definitely a good resource to families”* (service provider SP03).

Several participants mentioned that the weblink provided families with information that their child’s development was on track which reassured them:

*“If you do that screen and then you know that oh, your kid is not late or something…It is just a normal kid, like the other kids… So that way you know that you can feel rest assured”* (family member FA02).

### Barriers

#### Difficulties with accessibility

Some participants discussed how a limitation to the weblink was that it could not be accessed by parents who spoke certain languages as WMG-E was available only in four most commonly used languages for the area. Participants commented that many parents might not know about the weblink and recommended increasing advertising and communication about the weblink, such as through GP clinics:

*“Potentially some families are still not aware of [the weblink], and it's something that we could improve on”* (service provider SP03).

Some participants described how technology issues might hinder weblink use, including the slow loading time on the weblink, not having a mobile telephone, or not having access to the internet:

*“Not everyone is able to do things online”* (family member FA31).

In these cases, the service provider who introduced the family to the weblink might support them to complete it. The extended waiting periods for services might also deter families from following through with the self-referral process:

*“There's still the problem that they might self-refer but there’s, for more specialised services such as allied health, an extensive waiting list”* (service provider SP04).

Another barrier to accessing the weblink was the exclusion criteria, such as the age of the child in this specific project as six months to three years:

*“Maybe broaden the program for [children] a little bit older”* (family member FA60).

#### Absence of immediate clinician involvement during developmental screening

Some service providers and service navigators had concerns that the absence of immediate professional oversight in the weblink might contribute parent misunderstanding and anxiety about children’s developmental stages. This was especially among parents with limited knowledge of child development:

*“I think without a clinician’s clinical judgement at that time potentially some parents could perceive that information incorrectly or it could cause a little bit of anxiety in parents”* (service provider SP03).

Some service providers and navigators commented that digital feedback might not be appropriate when risk is involved, such as domestic violence or mental health problems:

*“The last lady that had domestic violence [DV], she would've got that report straightaway emailed to her. And my concern was when she didn't answer me, is that wherever the DV was coming from, they may have seen that”* (service navigator 1).

Some service providers and navigators mentioned items on the survey that could be improved or that parents did not understand what a certain question was asking:

*“I find out the most problem is that parents have different understanding of the questionnaire... One week ago, another mum there's item in 12 months check said can your, can your child speak mama or dad? So the child can speak mother and da, but cannot speak the full mum and dad. And the mum called me and said could you help me to pick an appointment with a speech pathologist? Because my daughter cannot speak”* (service navigator 2).

**Research Question 3: **What roles do service navigators play in facilitating engagement with the WMG-E program and improving service access and uptake of recommendations, and what challenges and strategies do they encounter in a multicultural community context?

## Theme 3.0: Enablers and barriers of Service Navigator implementation

### Enablers

#### Enhances uptake of family support services

Several participants described the helpfulness of the resources and service information provided by the service navigator:

*“[Service navigation] actually helps families get connected to what needs to be connected to, whether it’s within the community or whether it is getting help for a mum or for things that you help with your family”* (family member FA08).

The service navigator was described as playing a critical role in facilitating help-seeking, particularly for people who might not usually seek assistance:

*“Not everybody knows the service system or how to access support, so I think that if there’s a tool that’s able to guide them to where they need to go, then absolutely I think that it’s beneficial and have a place and help families that ideally would not always seek assistance”* (service provider SP02).

Participants discussed how the service navigator simplifies the dense and rapidly changing landscape of services by serving as a central point of contact:

*“I think always when services or families have one point of contact, it becomes far easier and far less overwhelming for them to navigate a service system”* (service provider SP02).

Support provided by service navigators, including regular check-ins and connecting families to services, was beneficial to families:

*“She just kind of like explained what the services that were available and some information about the services that …and she was quite helpful with offering services and trying to help me see what could assist me in my situation”* (family member FA60).

### Barriers

#### Participants’ narrow scope of engagement with offered services

In some cases, families interacted with services primarily for financial assistance despite referral for a diverse set of needs:

*“Only two families which both of them only contacted us to get some financial support; other than that they did not engage in other services like I tried to do an assessment with them to try and explore more of further issues but they did never engage”* (service provider SP01).

Service providers and navigators shared how multicultural families are sometimes slow to warm up to and trust the service navigators, which can be frustrating for service navigators:

*“They are migrants or they cannot speak English. Well of course they are not quite familiar with the local community services. And another thing is that it's really hard to engage them in the first few rounds”* (service navigator 1).

Once a relationship was established, families wanted to engage and receive support from service navigator.

### Theme 4.0: Suggested improvements for the service navigator role

#### Importance of providing accurate information about services

There was a risk that family expectations might differ to what services could provide and when the services could be provided. This was partly due to miscommunication between service navigators and services:

*“I am still waiting a hearing test because [my son] was referred for a hearing test”* (family member FA08).

One service provider recommended that service navigators need to have a thorough understanding of all the relevant services to reduce miscommunication:

*“Having a good understanding of what services provide. I guess meeting expectations… If information about our service is given by a third party, such as a service navigator, sometimes there can be a miscommunication”* (service provider SP04).

#### Importance of clarifying the service navigator role

Service providers and service navigators discussed how service navigators need to be clear with families about their role, particularly that the role did not include the provision of clinical advice:

*“When I first got connected with the family, I told them I'm not a clinical professional… I'm here to provide service linkage. So I'm not a clinical background because some mums just use me as a clinician”* (service navigator 2).

Service navigators might also need to specify how long they can support families so that families do not always depend on them, and that they can support other families:

*“Maybe [the service navigator] needs to say, okay, we're with you for this period of time. This is what I'm going to do, and this is what you need to do. And that's it. Make them take a bit of responsibility”* (service navigator 1).

Service providers suggested that services have a single individual designated to the service navigator role:

*“If her role is only focused on service navigation, I think the intervention will even be more successful”* (service navigator 2).

Service providers discussed how service navigators might not have capacity to support many families at one time. Some service providers and navigators did not think it was feasible to have a service navigator at all sites and recommended focussing on the vulnerable populations:

*“I don’t think that [service navigation is] always necessary... you don’t want to capture a bunch of people that actually don’t need it and they’re quite resilient. So I would kind of be capturing a very small percentage of people that you identify as having a potential underlying vulnerability”* (service provider SP02).

One service navigator suggested there could be different service navigator models depending on the needs of families:

*“We need to set up a different service navigation model for different types of participants in different local health communities based on the previous experience to save the time and also to set up the expectation and to release the pressure of the service navigator”* (service navigator 2).

## Discussion

The current study interviewed family members, service providers and service navigators to understand the enablers and barriers to implementing a WMG-E weblink alongside a service navigator in a multicultural community. Findings showed that the WMG-E weblink was perceived by families as easy to use, accessible by many families, and provided information to families regarding their child’s development. The service navigator role was highlighted as helpful in connecting families to appropriate services. Service providers appreciated the anticipatory guidance and awareness and information provided to families by the WMG-E platform. Specifically for the CALD community the language translations available on the weblink was regarded as particularly useful. This is consistent with previous findings about the need for language congruence when supporting families for completing developmental checks [21]. Barriers included that the weblink could not be accessed by some families, due to technology access issues. In the intervention group, this was overcome by the service provider who was introducing the family to the WMG-E link supporting families to complete the checks using the WMG-E. By utilising the service providers who are trusted and families are already engaged with, would be helpful in this regard. Other limitations to accessing the weblink included not having clinical oversight while families are completing the weblink at their homes or in the community and thereby families possibly misinterpreting the results or recommendations. However, it is to be noted that this may have been a unique issue during COVID-19 when services were closed. Also, the availability of Child and Family Hubs as a place-based ‘one-stop shop’ service that families can access and service navigators can refer to would be helpful in this regard [43].

The family members in this study highlighted that WMG-E is user-friendly, straightforward and quick to use. This contrasts with research showing that digital literacy is often poor and internet access is limited in CALD populations [44]. However, experience with mobile telephones facilitates digital literacy, suggesting that the family members who participated in this research may have been familiar with mobile phone use [44]. The simple questionnaire structure of the weblink may also have facilitated ease of use. For families with language issues, translations are available – but when low literacy or technological issues prevail, a trusted service provider could help a family to complete the weblink. This should form an important consideration during implementation as the families that WMG-E seeks to support often experiencing socioeconomic disadvantage and might not have the means to purchase technology. Future studies could investigate how families without access to technology could be involved in WMG-E, such as having an electronic device available to families when attending an in-person service. Further, language barriers were described by participants as another issue that requires attention. WMG-E was made available in four languages commonly used in the community where this study was conducted, and it is important to make provisions for those who speak languages other than the ones where translation was available.

According to the participating service providers and navigators, the WMG-E was limited by the absence of immediate clinician input when families completed the weblink, reflecting previous research [21–23, 45]. However, this may have been due to the COVID-19 related service closure when this study was conducted. The WMG-E weblink is intended to be completed by families in collaboration with a service provider the family trusts. Findings from this study highlighted the importance of implementing the WMG-E program with clinical follow-up and support when needs are identified, rather than completing the weblink in isolation. Service providers and navigators described limitations when electronic feedback is provided to families when risk is involved, and how families may become anxious if they do not understand the feedback. The therapeutic aspect of a clinician providing feedback to a family might be missed via electronic feedback [46]. Validity might also be an issue when families use a digital screening tool compared with a face-to-face assessment with a clinician [47]. Regarding risk, it is important for a protocol to be used when a family member has a positive screen for issues including mental health problems or domestic violence. For instance, contact information for domestic violence organisations should be made available to families where relevant, or a clinician could be alerted to the risk and an in-person appointment be organised, as recommended for other online interventions [48].

In this study, participants who are in the treatment group described the importance of the service navigator in directing families to services tailored to their needs on a continuous basis, reflecting previous research [30]. CALD populations may experience use of technology alone as impersonal [44]. A service navigator might provide the additional personal interaction needed to promote service uptake. Additionally, a service navigator might aid CALD families with low health literacy or communication difficulties by providing clear information about services that are available for their specific needs [1, 49]. Service providers highlighted the importance of making the service navigator role clear to manage families’ expectations, such as how long the service navigator might be able to support them. This might be particularly important for CALD communities as their expectations of services and health professionals may be unmet due to their previous experiences in their home countries [50]. In addition, service providers discussed how some families did not contact services that the service navigator recommended and focused on financial resources. This might be explained by the socioeconomic disadvantage experienced by families in this study [14]. It is possible that they focussed on meeting basic needs with limited capacity to address other needs, such as child developmental concerns or their own mental health challenges. The COVID-19 pandemic might have also reduced families’ capacity to access services [51]. More research is needed to understand the enablers of an effective service navigator, such as the skills or characteristics they require to ‘fit’ with the families they work with. Interestingly, families did not comment on the cultural background of the service navigator, and it was unclear whether the service navigator had the same cultural background as families. However, one participant highlighted that family members in CALD communities prefer support from providers with the same cultural background, as has been highlighted in prior research [1, 24]. While it might not be feasible to match clients’ and staff members’ cultural backgrounds, staff members should demonstrate cultural sensitivity. Further work is needed to increase the healthcare workforce with representation from CALD communities to provide culturally safe services to families from diverse communities and backgrounds [1, 24].

In addition, participants highlighted how WMG-E has the potential and capacity to increase families’ understanding about their child’s development, consistent with research on other screening applications used in mental health [52]. WMG-E might be especially important to increase knowledge of developmental milestones in CALD communities where barriers to education or information might exist [21, 23]. Future research is needed to ascertain the impact of WMG-E in increasing knowledge about child development in multicultural communities.

Although family members can experience confusion and frustration when engaging with child and family health services [53], the positive feedback obtained in this study might suggest that service navigators facilitated the navigation process for participants in this study. Participants also discussed how services adapted to the COVID-19 pandemic, but how there were increases in referrals and child behavioural problems due to COVID-19. This might have been particularly relevant for CALD families who might prefer face-to-face support and be less likely to engage with telehealth services [54, 55].

### Implications for health policy and practice

- The use of digital screening is complemented by the service navigator’s role to intervene and act as quickly as possible to the individual circumstances and the specific child and family needs. Future policy should include service navigators as part of the public health workforce, specifically in child and family health, mental health and social care services so that CALD families in particular who have technology access issues or needing support for interpreting the results and recommendations could be supported. This will strengthen integrated care and continuity of care policy.
- While the inclusion of service navigators is one important approach, future co-design work is required. The service navigator role needs to be relevant from a cultural and language perspective. Therefore, co-designing the WMG-E and service navigator program with the CALD community is important. For instance, future work needs to incorporate more languages than the four used in this study or employ service navigators of a certain cultural background who speak languages other than English.
- This study also has implications for future research in public health measures to engage multicultural families in developmental checks. This could include increasing awareness among families and clinicians and other staff working in early childhood education and social service agencies about using opportunistic contacts that they have with families with preschool children about digital programs such as WMG-E so that it can be incorporated into their routine work.. Further, health literacy is required regarding the available health and social care services in the community, to leverage existing resources and to avoid duplication of services. Establishment of Child and Family Hubs as a one-stop-shop service for multicultural families will also be helpful so that engagement with services following referral and continuity of care can be ensured.

### Strengths and Limitations

This study is novel in evaluating a new digital developmental surveillance program in addition to linkages and supports provided via a service navigator commensurate with the needs identified. A strength is the project’s inclusion of family members with various ethnic backgrounds and the use of a digital platform with translations available to cater for multicultural families. In addition, the study has many practical implications for CALD families, including the role of service navigators in supporting service access appropriate to their individual needs. Another strength is that this study reached data saturation in that new codes or themes were not emerging and new data repeated previous data [56].

The convenience sampling strategy of this project is a limitation because participants were not randomly selected and therefore might present a bias. Family member participants were overall highly educated, with 60% having more than high school education and 60% who were competent in English language. Therefore, these participants might not be representative of all CALD community members.

## Conclusions

In the present study, family members, service navigators and service providers from a multicultural community provided their perspectives on the enablers and barriers to implementing WMG-E, a digital developmental screening and ongoing monitoring tool, alongside a service navigator to link families with the relevant services as per need. Findings showed that family members found WMG-E to be user-friendly and informative regarding child development. The findings also highlighted the critical next step of accessing relevant services for further assessment when parental concerns are identified regarding the developmental progress of the children. Service navigators were perceived positively as they connected families to services and increased service uptake, although families need close follow up to ensure uptake of recommendations and engagement with services offered to them. This study demonstrated that WMG-E with the addition of a service navigator may be an important approach to reach and engage multicultural families who would not have otherwise engaged with routine child and family health services and hence would have missed completing development checks for their children. Further support for those with complex psychosocial needs via Child and Family Hubs, where services are co-located and integrated, would enable them to access relevant early intervention and support services in one place.

### Supplementary Information

Supplementary Material 1.

Supplementary Material 2.

## Data Availability

Data from the current study will not be made available, as participants did not consent for their transcripts to be publicly released. Extracts of participant responses have been made available within the manuscript. Please contact the corresponding author, Professor Valsamma Eapen, for any data requests.

## Abbreviations

CALD: Culturally and linguistically diverse
CDC: Centers for Disease Control and Prevention
DV: Domestic violence
LTSAE: Learn the Signs Act Early
RCT: Randomised control trial
WMG-E: Watch Me Grow – Electronic
