# Supplementary material for: Family member and service provider experiences and perspectives of a digital surveillance and service navigation approach in multicultural context: a qualitative study in identifying the barriers and enablers to Watch Me Grow-Electronic (WMG-E) program with a culturally diverse community
Source: BMC Health Serv Res. 2024 Aug 24;24:978. doi: 10.1186/s12913-024-11397-y (PMC11344394; doi:10.1186/s12913-024-11397-y)
Supplement: Supplementary file 1 — Supplementary Material 1. [file 12913_2024_11397_MOESM1_ESM.docx]

**APPENDIX 1 – QUALITATIVE STUDY DATA COLLECTION GUIDE**

**WMG-E Qualitative Interview Guide – Parent/Caregiver**

**Project Title: A ‘Digital’ Support for Child and Family Health Services for developmental surveillance: Using the Watch Me Grow web-app to address a critical COVID-19 service delivery gap**

Questions

1. Are you/have you attended a local child and family health service(s) as part of your/your child’s routine care? If so, can you please tell me about your general experiences when attending your local child and family health service(s)? What are some of the services that you have received?
2. Did the COVID-19 pandemic and lockdowns impact how often you and your child attended appointments with your local child and family health service(s)? Did you have to adopt to telehealth consultations/appointments?
3. Have your child had a developmental check before? If so, who did it and why?
4. Can you please tell me about your experiences in using the WMG-E App as a digital support tool for screening your mental health and psychosocial wellbeing, as well as your child’s developmental check?

- Is it easy and straightforward to use?
- How long did it took you to complete the screening checks?
- Do you have any feedback about the online referral guidelines/information provided by the WMG-E App at the end?
- What do you perceive as the potential issues to parents/caregivers using the WMG-E App?
- Do you have any suggestions for improvements that could be introduced to this digital tool?
- Would you recommend other parents/caregivers with young children to use this App? Why/Why not?

**For intervention participants ONLY:**

1. After you complete the WMG-E App, do you remember what happen next?

- Were you contacted by a service navigator/project officer?
- Were you given any referral information or resources to link you to your relevant services?
- Were you able to receive the services & supportive care relevant to your and/or your child’s needs?
- How much follow-up was there from your service navigator in this process? Did this meet your needs?
- Were there any problems that got in your way at that time?
- Overall, was having the service navigator helpful/beneficial to achieve your targeted care needs?

1. Would you recommend local child and family health services, such as Child and Family Health Nurse clinics, refugee health services, supported playgroups, parenting groups, etc., have an in-house service navigator as part of their routine care model allocated for families, especially those classified as at-risk? Why/Why not?

**For ALL participants:**

1. Would you recommend the WMG-E App as a digital support tool to be used in local child and family health services, such as Child and Family Health Nurse clinics, refugee health services, supported playgroups, parenting groups, etc. as part of their routine health and wellbeing checks for all parents/caregivers and their children? Why/Why not?
2. Is there anything I have not asked about that you would like to share about your experience with your local child and family health services (if any), the WMG-E App, and the service navigator (for intervention participants)?

**WMG-E Qualitative Interview AND Focus Group Guide – Service Providers/Health Workers/Professionals**

**Project Title: A ‘Digital’ Support for Child and Family Health Services for developmental surveillance: Using the Watch Me Grow web-app to address a critical COVID-19 service delivery gap**

Questions

1. What is your role in the [service organisation name] you are working in? What was your role in assisting and driving the WMG-E research project?
2. Describe your experience in providing, conducting, coordinating or supporting a [mental health / psychosocial wellbeing / child developmental check or screening] services to your clients/families in your organisation.

- Can you give me an example or some examples of these experiences and tell me how that work out?
- When do you usually provide these services or do these work?
- Is this something you do routinely? Or not?
- What are some of the tools do you use?
- How do you work with other health workers/professionals or members within your service organisation?
- How do you see your role? Do you see your role as making an impact to the families/patients/clients that you serve?

1. Did the COVID-19 pandemic and lockdowns impact how you work with families? Did you have to adopt to telehealth consultations/appointments?
2. Describe your experiences being involved in the WMG-E App program. Do you have any feedback about the referral guidelines/information provided by the WMG-E App?
3. What do you perceive as the barriers/facilitators to using a digital platform, like the WMG-E App, for families as a screening tool to assess where they are/what they need? (You may want to show pictures/screenshots of the WMG-E App questionnaires).
4. Does the use of a digital tool, such as WMG-E App, with the utilisation of a service navigator to link intervention families (participants) to the relevant services a feasible combination/approach for parents/caregivers with young children? Explain.

- Does this implementation approach work in your organisation? Why/Why not?
- Are there any problems with this pathway? If so, what are they?
- What suggestions do you have to address this to make this pathway better?

1. Would you recommend the implementation of WMG-E App +/- Service Navigator approach to be embedded as part of routine health and wellbeing checks for all parents/caregivers and their children in YOUR organisation? Why/Why not?
2. Would you recommend the implementation of WMG-E App +/- Service Navigator approach to be embedded as part of routine health and wellbeing checks for all parents/caregivers and their children in OTHER local child and family health services, such as Child and Family Health Nurse clinics, refugee health services, supported playgroups, parenting groups, etc? Why/Why not?
3. What are your thoughts on using a digital support tool (such as an App) to conduct screen health and wellbeing (e.g. mental health, psychosocial wellbeing and child development) checks in the future?
4. Is there anything I have not asked about that you would like to share about your experience participating in the research program, your role, the WMG-E App, and the service navigator (for intervention participants)?
