# Supplementary material for: Family member and service provider experiences and perspectives of a digital surveillance and service navigation approach in multicultural context: a qualitative study in identifying the barriers and enablers to Watch Me Grow-Electronic (WMG-E) program with a culturally diverse community
Source: BMC Health Serv Res. 2024 Aug 24;24:978. doi: 10.1186/s12913-024-11397-y (PMC11344394; doi:10.1186/s12913-024-11397-y)
Supplement: Supplementary file 2 — Supplementary Material 2. [file 12913_2024_11397_MOESM2_ESM.docx]

**Supplementary File Table 2.** Standards for Reporting Qualitative Research Checklist

| **Index** | **Topic** | **Section(s)** | **Page No. in the main manuscript file** |
| --- | --- | --- | --- |
|  | Title and Abstract |  |  |
| 1 | Title | Title Page | 1-3 |
| 2 | Abstract | Abstract | 4-5 |
|  | Introduction |  |  |
| 3 | Problem formulation | Background | 6-9 |
| 4 | Purpose or research question | Background – Research Aim | 9-10 |
|  | Methods |  |  |
| 5 | Qualitative approach and research paradigm | Methods | 10-14 |
| 6 | Researcher characteristics and reflexivity | Methods – Data Analysis | 10 |
| 7 | Context | Methods – Study Context | 10-12 |
| 8 | Sampling strategy | Methods – Participants in this qualitative study | 12 |
| 9 | Ethical issues pertaining to human subjects | Methods – Interview recruitment and procedures | 13 |
| 10 | Data collection methods | Methods – Interview Recruitment and Procedures | 13 |
| 11 | Data collection instruments and technologies | Methods – Interview Recruitment and Procedures, Supplementary Table 1 | 13 |
| 12 | Units of study | Methods – Characteristics of participants involved in this qualitative study  Methods – Participant Recruitment and Interviews | 12-13 |
| 13 | Data processing | Methods – Data Analysis, Figure 1 | 14 |
| 14 | Data analysis | Methods – Data Analysis | 14 |
| 15 | Techniques to enhance trustworthiness | Methods – Data Analysis | 14 |
|  | Results |  |  |
| 16 | Synthesis and interpretation | Results, Figure 2 | 15-28 |
| 17 | Links to empirical data | Results | 15-28 |
|  | Discussion |  |  |
| 18 | Integration with prior work, implications, transferability, and contribution(s) to the field | Discussion | 28-33 |
| 19 | Limitations | Discussion – Strengths and Limitations | 33 |
| 20 | Conclusion | Conclusion | 33-34 |
|  | Other |  |  |
| 21 | Conflicts of interest | Competing Interests | 35 |
| 22 | Funding | Funding | 35 |

**Reference:** O’Brien BC, Harris IB, Beckman TJ, *et al.* Standards for reporting qualitative research: a synthesis of recommendations. *Acad Med.* 2014;89:1245–51.
